# A laparoscopic treatment of a suspicious adrenal mass revealing an unsual cause of adrenal incidentaloma

**DOI:** 10.11604/pamj.2019.34.28.18239

**Published:** 2019-09-12

**Authors:** Houda Salhi, My Youssef Alaoui Lamrani, Nawal Hammas, Ousadden Abdelmalek, Hanan El Ouahabi

**Affiliations:** 1 Department of Endocrinology and Diabetology, University Hospital of Fez, Fez, Morocco; 2 Department of Radiology, University Hospital of Fez, Morocco; 3 Department of Pathology, University Hospital of Fez, Morocco; 4 Department of General Surgery, University Hospital of Fez, Morocco

**Keywords:** Adrenal Incidentaloma schwannoma, adrenalectomy, Adrenal Incidentaloma schwannoma, adrenalectomy

## Abstract

Schwannomas are rare tumors, usually benign, originating from the Schwann sheath of the peripheral or cranial nerves. They are an extremely uncommon cause of adrenal incidentaloma. It is difficult to diagnose adrenal schwannoma preoperatively by imaging alone due of their aspect suspect on it. We report a case of adrenal mass discovered incidentally in a 41 years old patient who presented an atypical abdominal pain. Biological assessment was unremarkable, and imaging studies did not show strict imaging criteria for a typical adenoma. A surgical excision of the adrenal incidentaloma was decided. Histopathological examination and immunohistochemical staining of the excised specimen revealed a benign schwannoma.

## Introduction

Schwannomas are unfrequent tumors, usually benign, originating from the Schwann sheath of the peripheral or cranial nerves [1]. Visceral schwannomas are extremely rare. Retroperitoneal schwannomas account for only 1-3% of all schwannomas and for only 1% of all retroperitoneal tumors [2]. They are usually discovered serendipitously. Herein; we report the case of an adrenal schwanoma discovered during a surgery of an adrenal incidentaloma suspected of malignancy.

## Patient and observation

**Figure 1:**
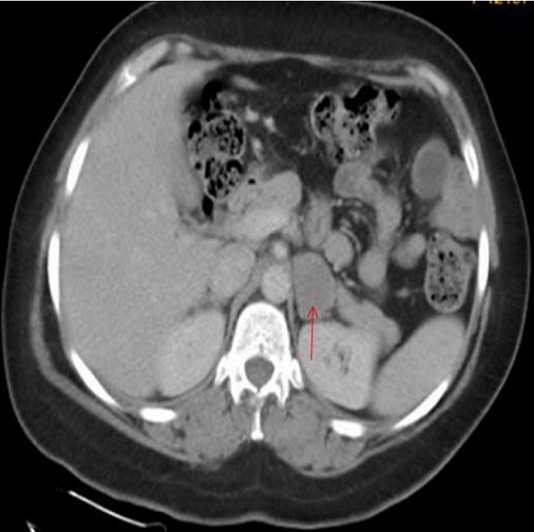
CT showed a hypodense left adrenal lesion measuring 2.5 cm X 3.4 cm in diameter with a spontaneous density of 20UH

**Figure 2:**
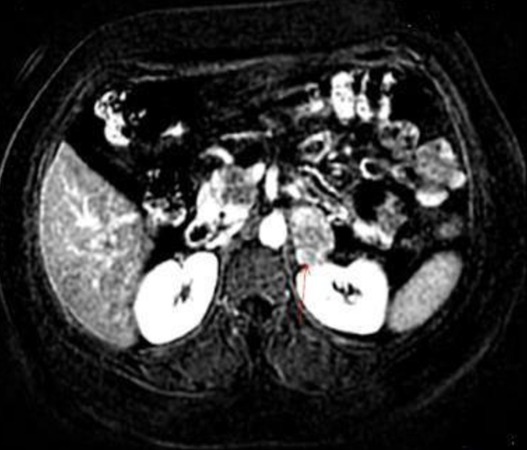
Magnetic resonance imaging revealed a round mass in the left adrenal gland. The mass registered low-signal intensity on the T1-weighted image, and a slightly high signal intensity on the T2-weighted image

**Figure 3:**
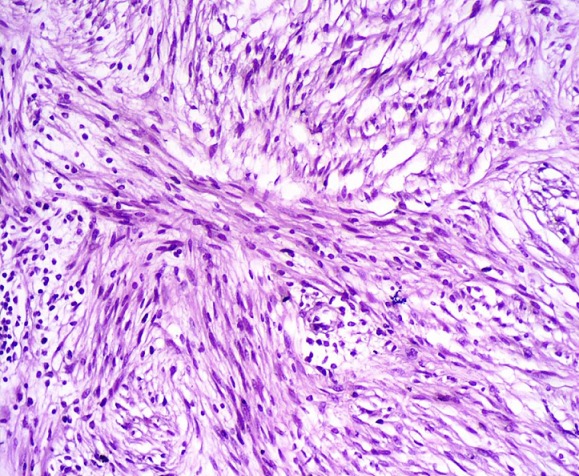
The tumor cells are fusiform, devoid of atypia (HES X 200)

**Figure 4:**
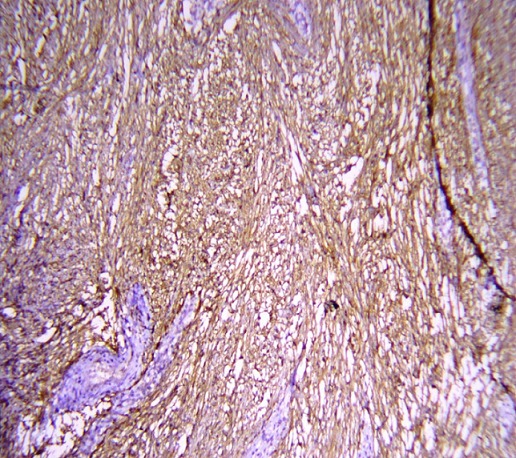
The tumor cells expressed PS 100

A 41 years old patient, presented with epigastric pain and indigestion since 1 year. Physical examination and ultrasound abdomen were normal. A symptomatic treatment was prescribed without improvement. On physical examination the abdomen was soft, blood pressure was 130/85 mm Hg and pulse rate was 72 beats/min; sinus rhythm was normal. Blood count and biochemistry analysis were within normal limits. Computed tomography of the abdomen showed a left adrenal mass (2.5 X 3.4 cm); with a spontaneous density of 20 Hounsfield units (UH). The relative and absolute washouts were 10 % and 16 % respectively (Figure 1). Based on a density of 20 UH, the mass did not meet strict CT criteria for diagnosis of adenoma. Therefore, MRI was indicated for additional tumor characterization. It showed a left adrenal, well limited; with low-signal intensity on the T1-weighted image, and slightly high-signal intensity on the T2-weighted image; moderately enhanced after contrast. The calculation of the signal drop on the in-phase and out-of-phase sequences shows a value of less than 10% (Figure 2). The metabolic workup, including serum electrolytes, urinary metanephrine, was within the normal range. Also, a dexamethasone suppression test showed values within the normal range. Due to the suspect nature of the masse; a left adrenalectomy was indicated, which was performed via a transperitoneal laparoscopic approach. The histopathological and immunohistochemical study revealed the presence of a well-limited tumor of 3.3 X 2.9 cm. The tumor cells expressed PS 100 (Figure 3, Figure 4). They did not express AML; the desmin; synaptophysin; chromogranin A or CD 117. This aspect is in favor of a benign tumor schwannoma type. The postoperative recovery was uneventful. A long term CT scanner follow-up at 6, 12, and 18 months will be necessary to get a recurrence.

## Discussion

The greater number of the tumors originating in the adrenal medulla is pheochromocytoma, neuroblastoma, or ganglioneuroma. Adrenal schwannoma is rarely encountered, even though it similarly originates from the cells of the neural crest. Its first description was done in 1908 by Verocay as neurinoma [3]. In 1932; Masson proposed the terminology of Schwannoma [4]. This tumor is more common in women with an approximate male-to-female ratio of 2:3 [5]. Given the nonfunctional nature of schwannoma, only positive hormonal studies, such as elevated urine metanephrines in most cases of pheochromocytoma, can unequivocally exclude the diagnosis of schwannoma [6]. In our case, the urinary metanephrine and overnight dexamethason suppressive test were within the normal range. The diagnosis of adrenal schwannoma is often incidental, because slow growing, deeply situated tumor. It is usually present for long periods of time before patients complain of symptoms [7]. However, some patients may present with atypical symptoms such as: abdominal or lumbar pain, hematuria related to compression of neighboring organs [8]. It is difficult to diagnose adrenal schwannoma preoperatively by imaging alone and surgical resection is the primary management. On computed tomography, a schwannoma can appear like a round or oval mass that may be homogeneous, as in the case we present [9]. However, Due to the frequent degeneration, cystic change, hemorrhage and calcification inside this masse they often appear heterogeneous on imaging. The mass demonstrated variable homogeneous or heterogeneous enhancement with addiction of contrast arising the difficulty to differentiate between malignant or benign schwannoma [10] this was the case of our patient. MRI imaging findings are nonspecific. Adrenal schwannoma is of low signal intensity on T1WI and heterogeneously high intensity on T2WI on MRI [11]. Intratumoral cysts were found in 63% of cases for benign schwannomas and in 75% of cases for malignant schwannomas. This sign is exceptional in other peritoneal tumors [7]. By cause of that a schwannoma is diagnosed only in one third of cases by imaging, the definitive diagnosis must be determined through histologic means as seen in our patient [12]. Schwannoma malignant form is frequently associated with von Recklinghausen disease and neurofibromatosis [13]. Therefore, it is very important to remove the tumor completely surgically. In cases of small tumors (<4 cm) as our case, the laparoscopic resection can be preferred to conventional approach. Conventionally, schwannomas present as alternating compact areas termed Antoni A and loosely textured paucicellular areas described as Antoni B [14]. These 2 patterns may coexist in the same specimen but usually one is predominant [8]. Immunohistochemical stains for the neuroectodermal marker S100 are nearly universally positive in these tumors [15] but they stain negative for CD117, desmin, CD34, HMB45, synaptophysin, cytokeratin and smooth muscle acting on immunohistochemistry [16] as it seen in our case.

## Conclusion

The present case suggests that a preoperative diagnosis of adrenal schwannoma is difficult. Surgical excision can provide the benefit of a definitive diagnosis which can only be made by histological and immunohistochemical evaluations.

## Competing interests

The authors declare no competing interests.
